# EZH2 promotes the expression of LPA1 by mediating microRNA-139 promoter methylation to accelerate the development of ovarian cancer

**DOI:** 10.1186/s12935-020-01622-z

**Published:** 2020-11-16

**Authors:** Dongbo Wu, Fanglan Wu, Birong Li, Wei Huang, Donglian Wang

**Affiliations:** 1 Department of Obstetrics and Gynecology, The First Hospital of Changsha, Changsha, 410000 People’s Republic of China; 2 Department of Clinical Laboratory, The First Hospital of Changsha, Changsha, 410005 People’s Republic of China; 3 Department of Gynecology, Hunan Provincial People’s Hospital (The First Affiliated Hospital of Hunan Normal University), No. 61, Western Jiefang Road, Changsha, 410000 Hunan People’s Republic of China; 4 Department of Gynecology, The Maternal and Child Health Hospital of Hunan Province, Changsha, 410000 People’s Republic of China

**Keywords:** Ovarian cancer, Tumor growth, Enhancer of zeste 2 polycomb repressive complex 2 subunit, microRNA-139

## Abstract

**Background:**

It has been known that ovarian cancer (OC) is a leading cause for women mortality globally. We aimed to analyze the underlying mechanism supporting that enhancer of zeste homolog 2 (EZH2) affected the development of OC *via* the involvement of microRNA-139 (miR-139)/transforming growth factor beta (TGF-β)/lysophosphatidic acid-1 (LPA1) axis.

**Methods:**

High expression patterns of EZH2 and miR-139 and low LPA1 expression pattern in OC were evaluated using RT-qPCR and immunoblotting, while their correlation was assessed by the Spearman’s rank and Pearson’s correlation coefficient. Subsequently, dual-luciferase reporter gene assay was applied to validate the binding relationship between miR-139 and LPA1, while H3K27me enrichment was assessed by ChIP assay. After that, the effects of altered expression of EZH2, miR-194, or LPA1 on the cell biological functions and the expression pattern of TGF-related factors were evaluated.

**Results:**

We found that EZH2 repressed the miR-139 expression pattern by recruiting H3K27me3 to promote miR-139 promoter methylation, while silencing of EZH2 suppressed in vitro cancer progression by increasing miR-139. LPA1 was a target of miR-139, and could activate the TGF-β signaling pathway, which hastened the OC progression. miR-139-targeted inhibition of LPA1 and LPA1-activated TGF-β signaling pathway were evidenced to be critical mechanisms underlying the effects of EZH2 on OC cells. Lastly, silencing of EZH2 inhibited the xenograft growth in vivo.

**Conclusions:**

EZH2 could down-regulate miR-139 expression pattern by recruiting H3K27me3 to promote the miR-139 promoter methylation and activate the TGF-β pathway by up-regulating LPA1, which contributed to the progression of OC. The current study may possess potentials for OC treatment.

## Background

Ovarian cancer (OC), a prevalent type of cancer affecting women, was causative of 184,799 deaths globally in 2018 [1]. On the basis of OC pathogenesis and prognosis, the disease can be categorized into five subtypes: high-grade serous, clear-cell, endometrioid, and mucinous, as well as low-grade serous cancer [2]. Due to the intricate etiology of OC influenced by numerous factors, various corresponding therapies were developed, however the survival rates did not change significantly due to unsatisfactory treatment outcomes [3]. In this case, obtaining a comprehensive understanding of the process and the molecular mechanisms regarding OC development and identifying novel markers for OC treatment are of crucial importance [4].

Enhancer of zeste homolog 2 (EZH2) has been recurrently implicated in OC. For instance, EZH2 in cooperation with long non-coding RNA (lncRNA) LINC00702 could radically regulate OC progression [5]. An existing study unraveled that EZH2 played a crucial role in the development of OC [6]. EZH2 was also reported to affect tumorigenesis by mediating the methylation of microRNA (miR)-211 [7]. Besides, EZH2 was validated to meditate and influence the insulin-like growth factor 1 receptor by direct transcriptional suppression of miR-139 [8]. The aberrant forms of miR-139 were further implicated in OC, for instance, miR-139-3p was associated with the time of progression in epithelial OC [9], and miR-139-5p was reported to reduce cisplatin-resistance in OC [10]. Besides, miR-139-3p is recently proposed to function as a tumor suppressor in OC [11]. Furthermore, an existing study documented the involvement of miR-139-5p in respect to OC tumor staging and grading among a total of 583 miRNAs based on the oncomiR database, and miR-139 was found to participate in OC [12]. Interestingly, it was identified that lysophosphatidic acid 1 (LPA1) as a viable target of miR-139 through online predication on the TargetScan database. LPA was initially elevated in patients with OC manifesting ascites and activates ovarian and breast cancer cells [13]. LPA1 was also associated with OC cell migration and may prolong the metastatic progression in OC [14]. The correlation between LPA1 and the transforming growth factor beta (TGF-β) has been investigated previously [15].

On the basis of the abovementioned literature, we hypothesized that EZH2/miR-139/LPA1 axis played a vital role in OC development and we aimed to understand the molecular mechanisms of OC and provide a clinical insight for OC.

## Materials and methods

### Ethics statement

Informed written consents were collected from all participants prior to enrollment. All experiments were carried out with approval of the Ethics Committee of the Hunan Provincial People’s Hospital, and in strict accordance with the principles stated in the Helsinki Declaration.

### Bioinformatics analysis

OC-related microarray dataset GSE54388 was acquired from the Gene Expression Omnibus (GEO) database (https://www.ncbi.nlm.nih.gov/gds). Besides, the platform GPL570 was adopted for analyzing this microarray, which included 22 samples (6 normal samples and 16 OC samples). The differentially expressed genes relevant to OC were analyzed using the R package “limma” (screening threshold: log Foldchange > 1.5; *p* < 0.01). Finally, the TargetScan database was applied for the prediction for downstream target gene of the miRNAs.

### Study subjects

OC tissue sample and adjacent normal tissue sample (at least 5 cm away from the primary tumor) were harvested from 69 female patients who underwent treatment from October 2010 to October 2013 in the Hunan Provincial People’s Hospital. The patients aged from 42 to 75 years old with a median age of 55 years. OC type distribution was as follows: 30 cases of epithelial OC, 19 cases of serous cystadenocarcinoma, 13 cases of mucinous cystadenocarcinoma and 7 cases of the other subtypes of OC. No patients were instilled chemotherapy or radiotherapy. Two experienced pathologists confirmed all tissue specimens. The OC tissues were harvested from the primary tumors. All harvested tissues were immediately stored in liquid nitrogen or fixed in 10% formaldehyde, which were then paraffin-embedded. The follow-up for the enrolled patients expanded over for 5 years, with a mean survival time of 36.98 months. The survival rate was calculated using the Kaplan-Meier method. From the statistical data, a 5-year survival rate of 34.78% was obtained. The demise of a patient was considered as the end point of follow-up. The overall survival (OS) is defined as the day of undergoing resection surgery to the day of patient death.

### Immunohistochemistry

The formalin-fixed, paraffin-embedded human OC tissues were used for immunohistochemistry examination. After xylene dewaxing and gradient alcohol dehydration, the cells were incubated with 3% H_2_O_2_. Following the antigen retrieval, the sections were blocked using the normal goat serum solution and subsequently incubated with the primary antibodies (Abcam Inc., Cambridge, UK) at 4℃ overnight, including EZH2 (ab195409, 1:100), LPA1 (ab166903, 1:80), TGFβ1 (ab92486, 1:100), phosphorylated Smad3 (ab52903, 1:200) and phosphorylated Smad2 (ab188334, 1:100). After that, the sections were incubated with the secondary antibody to Immunoglobulin G (IgG; ab150083, 1:100). The phosphate buffer solution (PBS) was used as a negative control (NC) for the primary antibody. Finally, the average number of positively stained cells in five randomly selected fields was microscopically observed. The density of the positive cells could be semi-quantitatively graded according to the percentage of the positive cells: positive cells < 15% are negative (0), 15–25% are (+), 25–50% are (++), 50–75% are (+++), > 75% are (++++).

### Cell culture

Normal human ovarian epithelial cell line (IOSE80) and human OC cell lines (HO8910, SKOV3 and ES2) from the Chinese Academy of Sciences cell bank (http://www.cellbank.org.cn) were obtained. All cells were cultured using the Dulbecco’s modified Eagle’s medium (DMEM; 12,800,017, Gibco, Carlsbad, CA, USA) supplemented with 10% fetal bovine serum (FBS, 26,140,079, Gibco) and 1% penicillin/streptomycin. After 24 h of incubation in a humidified incubator (BB15, Thermo Fisher Scientific, Waltham, MA, USA), the medium was renewed. The subculture was performed every 72 h. The cells were harvested to detect the expression pattern of EZH2 by reverse transcription quantitation-polymerase chain reaction (RT-qPCR) and western blot analysis, from which the cell line with the highest expression of EZH2 was selected for further analysis.

### Cell transfection and grouping

Cells were seeded in a 6-well plate 24 h before transfection using Lipofectamine 2000 (20 µL; 11,668,019, Thermo Fisher Scientific). The plasmid and Lipofectamine 2000 were diluted in 500 µL of serum-free medium, gently mixed, and cultured. The mixture was transferred to the 6-well plate with 500 µL of mixture in each well. After 8 h, the cells were further incubated in penicillin/streptomycin-free DMEM with 10% FBS for 48 h. For transfection, the cells were treated in different groups: shRNA-negative control (NC) group (transfected with NC shRNA), shRNA-EZH2 group (transfected with shRNA against EZH2), oe-NC group (transfected with EZH2 overexpression NC plasmid), oe-EZH2 group (transfected with EZH2 overexpression plasmid), oe-EZH2 + DMSO group (transfected with EZH2 overexpression plasmid and treated with DMSO), oe-EZH2 + DZNep group (transfected with EZH2 overexpression plasmid and treated with a histone methyltransferase inhibitor, DZNep), mimic-NC group (transfected with mimic NC), miR-139 mimic group (transfected with miR-139 mimic), miR-139 mimic + oe-LPA1 group (transfected with miR-139 mimic and LPA1 overexpression plasmid), sh-NC group (transfected with NC shRNA), sh-LPA1 group (transfected with shRNA against LPA1), sh-LPA1 + DMSO group (transfected with shRNA against LPA1 and treated with DMSO), sh-LPA1 + TGF-β1 group (transfected with shRNA against LPA1 and subjected to 2 h incubation of 10 ng/mL TGF-β1), shRNA-EZH2 + inhibitor-NC group (co-transfected with shRNA against EZH2 and inhibitor NC), shRNA-EZH2 + miR-139 inhibitor group (co-transfected with shRNA against EZH2 and miR-139 inhibitor), shRNA-EZH2 + oe-LPA1-NC group (transfected with shRNA against EZH2 and LPA1 overexpression NC plasmid) and shRNA-EZH2 + oe-LPA1 group (co-transfected with shRNA against EZH2 and LPA1 overexpression plasmid). RT-qPCR was then conducted to regulate the efficiency of shRNA (shRNA-EZH2*1, shRNA-EZH2*2, shRNA-EZH2*3, sh-LPA1*1, sh-LPA1*2, sh-LPA1*3), and shRNAs with the highest interference efficiency were selected for subsequent experimentation. The full-length cDNA sequences of EZH2, miR-139, and LPA1 (from the Ensembl database) and their respective NC nonsense sequences were designed using the lentiviral vector design software (Ambion, Austin, TX, USA). All plasmids, vector construction, sequencing identification, virus packaging and titer detection were provided by the Shanghai Genechem Co., Ltd. (Shanghai, China).

### RNA isolation and quantification

The total RNA content from OC tissue or cells was extracted using TRIzol (15596-018, Beijing Solarbio Life Sciences Co., Ltd., Beijing, China) after which its concentration was determined. Then, complementary DNA was synthesized by a reaction of 2 µg RNA with the TaqMan reverse transcription reagent (Roche Diagnostics, Indianapolis, IN, USA) at 42 °C for 50 min, which was followed by amplification of the target gene fragment by PCR (50 µL reaction system). The corresponding primers were synthesized by the Sigma-Aldrich Chemical Company (St Louis, MO, USA) (Table 1). Glyceraldehyde-3-phosphate dehydrogenase (GAPDH) and U6 served as the loading controls. The relative expression of the target gene was calculated based on the 2^−ΔΔCT^ method.

**Table 1:** Primer sequences for RT-PCR

Gene	Primer sequence (5′–3′)	
EZH2	F: TTGTTGGCGGAAGCGTGTAAAATC	R: TCCCTAGTCCCGCGCAATGAGC
miR-139	F: UGGAGACGCGGCCCUGUUGGAG	R:CAAACCAAAGATAAACGTGGATT
LPA1	F: ATCGGGATACCATGATGAGTC	R: TCCGTTCTAAACCACAGAGT
U6	F: GCTTCGGCAGCACATATACTAAAAT	R: CGCTTCACGAATTTGCGTGTCAT
GAPDH	F: TGAACGGGAAGCTCACTGG	R: TCCACCACCCTGTTGCTGTA

EZH2, enhancer of zeste homolog 2; miR-139, microRNA-139; LPA1, lamina-associated polypeptide 1; GAPDH, glyceraldehyde-3-phosphate dehydrogenase; F, forward; R, reverse

### Western blot analysis

In strict accordance with the provided instructions, the total protein content from the cells or tissues was extracted using pre-cooled radioimmunoprecipitation assay (RIPA) lysis buffer containing phenylmethylsulfonyl fluoride (R0010, Beijing Solabio Life Sciences Co., Ltd., Beijing, China). The protein concentration was determined using a bicinchoninic acid (BCA) kit (20201ES76, Yeasen Biotechnology Co. Ltd., Shanghai, China). Then equal amounts of protein was separated by a regimen of sodium dodecyl sulfate-polyacrylamide gel electrophoresis and then transferred onto a polyvinylidene fluoride membrane (Millipore Corp, Billerica, USA) by the wet transfer method. After a membrane blockade using 5% bovine serum albumin, the membrane was incubated with the following diluted primary antibody: rabbit antibodies to EZH2 (ab195409, 1:1000), LPA1 (ab166903, 1:3000), CyclinD1 (ab134175, 1:3000), CDK2 (ab32147, 1:5000), MMP-2 (ab37150, 1:2000), MMP-9 (ab73734, 1:2000), TGF-β1 (ab92486, 1:5000), Smad3 (ab40854, 1:5000), p-Smad3 (ab52903, 1:2000), Smad2 (ab40855, 1:5000), p-Smad2 (ab188334, 1:5000), ERK (ab17942, 1:1000), p-ERK (ab201015, 1:1000), Jnk (ab179461, 1:1000), p-Jnk (ab124956, 1:1000), p38 (ab170099, 1:2000), p-p38 (ab4822, 1:1000), Cleaved Caspase-3 (ab49822, 1:500), Bax (ab32503, 1:2000), Bcl-2 (ab59348, 1:1000) and GAPDH (ab181602, 1:10,000). Horseradish peroxidase-labeled goat anti-rabbit secondary antibody IgG (ab6721, 1: 5000) was then incubated with the membrane. All antibodies were acquired from Abcam (Cambridge, UK). Protein bands were quantitated using the QuantityOnev 4.6.2 software. The relative protein expression was expressed as the ratio of the gray value of the corresponding protein band to that of the GAPDH band.

### Dual luciferase reporter gene assay

The wild type (WT) sequence of the LPA1 mRNA 3’-untranslated region (UTR) containing the putative binding site and the mutant type (MUT) sequence obtained by site-directed mutation were synthesized. Next, the pmiR-RB-REPORTTM plasmid (Guangzhou RiboBio Co., Ltd., Guangzhou, Guangdong, China) was subjected to restriction endonuclease cleavage. The synthesized fragments WT and MUT were incorporated into the pmiR-RBREPORTTM vectors (Guangzhou RiboBio Co., Ltd., Guangzhou, Guangdong, China) with the empty plasmid serving as a NC. The correctly sequenced luciferase reporter plasmids WT and MUT were reserved for subsequent transfection. The vector containing MUT and WT co-transfected into the Human embryo kidney 293T cells with mimic-NC or miR-139 mimic, respectively. After 48 h of transfection, the cells were lysed and centrifuged for 3 to 5 min. The relative light unit was detected in the dual luciferase reporter assay system (Promega Co, Madison, WI, USA). The relative luminescence activity was described as the ratio of the firefly luciferase relative light unit (RLU) to the renilla luciferase RLU using a Renilla luciferase assay kit (YDJ2714, Shanghai Yudu Biotechnology Co., Ltd., Shanghai, China).

### Chromatin immunoprecipitation (ChIP) assay

The enrichment of H3K27me3 in the miR-139 promoter was investigated using the ChIP kit (Millipore Corp, Billerica, MA, USA). Cells were fixed with 1% formaldehyde, followed by ultrasonication (10 s on, 10 s off, 15 pulses). After centrifugation at 13,000 rpm, the supernatant was collected and transferred to three tubes containing the positive control antibody, the negative control IgG antibody, the target protein specific H3K27me3 (ab192985, Abcam, Cambridge, UK), respectively. After overnight incubation at 4 °C, the endogenous DNA-protein complex was precipitated by the protein agarose/sepharose. Then the supernatant was removed after centrifugation, and the non-specific DNA-protein complex was rinsed. The crosslinking was reversed overnight at 65℃, and the DNA fragment was purified and extracted based on a phenol/chloroform procedure. The INPUT was used as the loading control, and the miR-139 gene promoter-specific primers are shown in Table 1.

### 5-Ethynyl-2’-deoxyuridine (EdU) assay

After 48 h of transfection, the OC cells were incubated in the EdU medium (100 µL/well) for 2 h, then fixed with 100 µL of the cell fixative for 30 min at room temperature. After incubation with 2 mg/mL glycine, the cells were permeabilized using PBS with 0.5% Triton-100. Next, the cells were reacted with 1× Apollo staining reaction solution in conditions devoid of light and permeabilized again, followed by a rinse with methanol. Lastly, 1× Hoechst 33,342 reaction solution was used to stain the cells for 30 minutes at room temperature. Under the microscope, six to ten visual fields were randomly selected and the number of EdU-positive cells (nucleus stained in red) was calculated. EdU labeling rate (%) = the number of positive cells (a) / (a + the number of negative cells) × 100%.

### Transwell assay

The Transwell chamber (Millipore Corp, Billerica, MA, USA) was pre-heated and placed in a 24-well plate. Serum-free DMEM and DMEM containing 20% FBS at a volume of 0.5 mL were separately added to the different chamber, and the chamber was hydrated in the incubator for 2 h. After detachment, 0.1 mL of the diluted cell suspension (5 × 10^4^ cells/mL) was added in the apical chamber, while 0.5 mL of the complete medium was loaded into the 24-well plate. After 24 h of incubation, the cells in the apical membrane were gently wiped off with cotton. The migrated cells were fixed using pre-cold paraformaldehyde for 30 min, stained with 1% crystal violet, and rinsed under running water. The migrated cells were observed under an inverted microscope (Olympus Optical Co., Ltd., Tokyo, Japan). In the invasion experiment, the equipment and procedures used were fundamentally similar as the migration experiments. In addition, Matrigel (BD Biosciences, San Jose, CA, USA) was required for the experiments. Matrigel and DMEM were mixed at a ratio of 1:5, and 300 µL of the mixture was added to the apical chamber and incubated in an incubator for 6 h. The development of a white layer was indicative of successful Matrigel preparation. The remaining steps were the same as the cell migration experiment.

### Flow cytometry

After 48-h transfection, the cells were detached using 0.25% ethylenediaminetetraacetic acid-free trypsin, and centrifuged two times. According to the provided instructions of the Annexin-V-fluorescein isothiocyanate (FITC) Apoptosis Detection Kit (556547, Shanghai Shuojia Biotechnology Co., Ltd., Shanghai, China), the Annexin-V/propidium iodide (PI) staining solution was prepared by combining Annexin-V-FITC, PI, N-2-hydroxyethylpiperazine-N’-2-ethanesulfonic acid (HEPES) buffer at a ratio of 1:2:50. A total of 1 × 10^6^ cells was resuspended in 100 µL of the staining solution, and then incubated for 15 min at room temperature, followed by reaction with 1 mL of HEPES buffer. The level of apoptosis was measured using a flow cytometer (Bio-RadZE5, Bio-Rad, Inc., Hercules, CA, USA). The FITC had a precise maximum absorption wavelength (488 nm) and excitation wavelength (525 nm). The maximum absorption and emission wavelengths of the PI-DNA complex were 535 nm and 615 nm, respectively.

### Establishment of tumor xenografts in nude mice

Thirty-two specific pathogen-free (SPF) nude mice (female; aged 4 weeks; weighing 20–22 g; Shanghai SLAC Laboratory Animal Co., Ltd., Shanghai, China) were employed for the animal experiments. The mice were divided into 2 groups (n = 16). The cells transfected with shRNA-NC or shRNA-EZH2 were detached using 0.25% trypsin, centrifuged and dispersed into a single cell suspension with normal saline. Cells (2 × 10^6^) were resuspended in 50 µL of normal saline, mixed with 50 µL of Matrigel Matrix (1:1), and seeded into the oxters of the nude mice. The nude mice were euthanized by an injection of 40 mg/kg pentobarbital on the 7th, 14th, 21st, and 28th days, respectively, after which the tumors were harvested. The tumors were weighed and the tumor volume was calculated. The tumor tissues were preserved, and embedded in paraffin for immunohistochemistry.

### Statistical analysis

Data from this study were analyzed using the SPSS 21.0 (IBM Corp. Armonk, N.Y., USA) statistical software. The measurement data were presented as mean ± standard deviation. For data conforming to the normal distribution and homogeneity of variance, the comparison between two groups was conducted by the unpaired *t*-test, while comparison of data in one group was conducted by the paired *t*-test. The comparison among multiple groups was analyzed by one-way analysis of variance (ANOVA) followed by the Tukey’s post hoc test, while data at different time points were analyzed by repeated measurement ANOVA, followed by the Bonferroni’s post hoc test. The rank sum test was performed if the normal distribution or the homogeneity of the variance was not met. Enumeration data were analyzed by the Chi-square test. The correlation between miR-139 and EZH2 was analyzed by the Pearson’s correlation coefficient. Survival rate of patients was calculated based on the Kaplan-Meier method, while the differential analysis was verified by Log-rank. In all statistical references, a value of *p* < 0.05 was indicative of statistically significant difference.

## Results

### EZH2, miR-139 and LPAR1 are potentially involved in OC progression

**Fig. 1:**
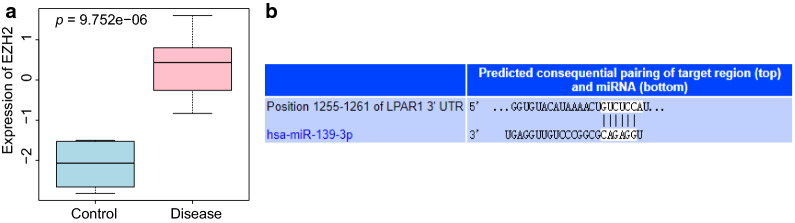
EZH2, miR-139 and LPAR1 may be involved in the progression of OC. **a** EZH2 expression pattern in the OC-related GSE54388 microarray in which the blue box in the left indicated normal samples while the red one referred to OC samples. **b** The binding sites between LPAR1 and miR-139 predicted by TargetScan

The differentially expressed genes in the microarray dataset GSE54388 obtained from the GEO database was screened while EZH2 was observed to function as a key highly expressed gene in OC (*p* = 9.752e−06; Fig. 1a). EZH2 was reported to function as an upstream regulator of miR-139-3p and could mediate the tumor-suppressive role of miR-139-3p [16]. Besides, LPAR1 was predicted as a target gene of miR-139 while the binding sites between miR-139 and LPAR1 were predicted using TargetScan (Fig. 1b). From the aforementioned findings, we inferred that EZH2, miR-139 and LPAR1 might participate in OC development.

### EZH2 is highly expressed in OC and indicates the poor survival

**Fig. 2:**
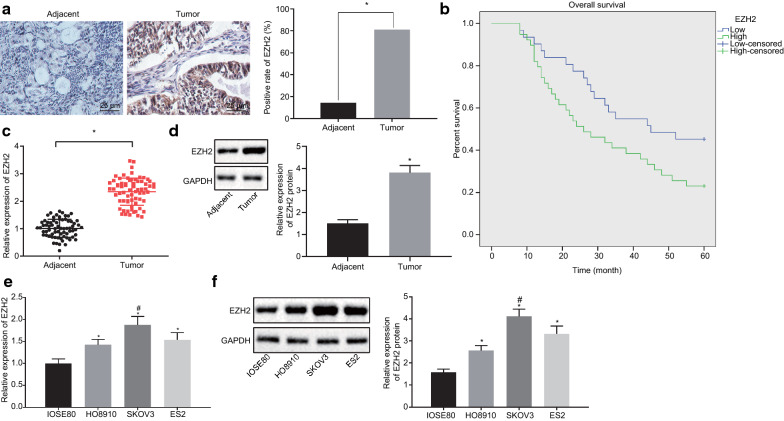
High expression pattern of EZH2 is associated with the prognosis of OC patients. **a** Immunohistochemical staining of EZH2 in clinical OC tissues and adjacent normal tissues (×400) (n = 69). **b** Survival analysis by Kaplan-Meier method. **c** the mRNA expression pattern of EZH2 in the OC and adjacent normal tissues as determined by RT-qPCR. **d** The protein expression pattern of EZH2 in the OC tissues and adjacent normal tissues as measured by Western blot analysis. **e** The mRNA expression pattern of EZH2 in the HO8910, SKOV3, ES2 and IOSE80 cells as determined by RT-qPCR. **f** The protein expression pattern of EZH2 in the HO8910, SKOV3, ES2 and IOSE80 cells as measured by Western blot analysis. *p < 0.05 vs. adjacent normal tissues or IOSE80 cells; # refers to the cell line with the highest expression pattern of EZH2. The measurement data were expressed as mean ± standard deviation. The paired-designed data was compared using the paired *t*-test. The one-way analysis of variance was adopted for comparison among multiple groups, followed by Tukey’s post hoc test. The experiment was conducted 3 times independently

To verify the EZH2 expression pattern in OC, immunohistochemistry was conducted. Results showed the positive expression pattern of EZH2 in brownish yellow, where EZH2 was expressed in the nucleus, cytoplasm and cell membrane. EZH2 expression in diseased tissues was much higher than that in the adjacent normal tissues (*p* < 0.05; Fig. 2a). Through analysis of the correlation between EZH2 and the clinicopathological features in 69 cases of OC, our findings revealed that the number of OC clinical specimens with a high EZH2 expression was significantly larger than that of OC clinical specimens with a low EZH2 expression pattern, and that the expression pattern of EZH2 was not correlated with the histological types of tumors and patient age, but was correlated with the histological grade, FIGO stage and lymph node metastasis (LNM) (*p* < 0.05; Table 2). Survival analysis based on the Kaplan-Meier method revealed that compared to patients with a high EZH2 expression pattern, the OS of patients with a low expression pattern of EZH2 was much longer (*p* < 0.05; Fig. 2b). RT-qPCR (Fig. 2c) and Western blot analysis (Fig. 2d) consistently showed that the expression pattern of EZH2 in the OC tissues was notably higher in contrast to the adjacent normal tissues (*p* < 0.05). The expression pattern of EZH2 in the 3 OC cell lines (HO8910, SKOV3 and ES2) and normal human ovarian epithelial cell line (IOSE80) was analyzed by RT-qPCR and Western blot analysis. The results revealed that EZH2 was expressed at a higher mRNA and protein level in the HO8910, SKOV3 and ES2 cell lines, compared with the IOSE80 cell line. In regard to the highest EZH2 expression pattern in SKOV3 cell line, the SKOV3 cells were selected for subsequent experimentation (*p* < 0.05; Fig. 2e–f). In summary, EZH2 is highly expressed in the OC tissues and cells, and a high expression pattern of EZH2 is associated with poor survival.

**Table 2:** Relationship between EZH2 expression and clinicopathological features of OC

Clinicopathological parameters	n	Expression of EZH2		*p*-value
		Poor expression (n = 31)	High expression (n = 38)	
Age				
≤ 50	46	21 (45.65%)	25 (54.35%)	> 0.999
> 50	23	10 (43.48%)	13 (56.52%)	
Histological classification				
Epithelial ovarian cancer	30	14 (46.67%)	24 (53.33%)	0.864
Serous	19	8 (42.11%)	15 (57.89%)	
Mucous	13	5 (38.46%)	9 (61.54%)	
Other	7	4 (57.14%)	3 (42.86%)	
Differentiation grading				
Highly-moderately	23	17 (73.91%)	6 (26.09%)	< 0.001
Poorly	46	14 (30.43%)	32 (69.57%)	
FIGO staging				
I–II	41	23 (56.10%)	18 (43.90%)	0.029
III	28	8 (28.57%)	20 (71.43%)	
Lymph node metastasis				
Yes	32	5 (15.63%)	27 (84.38%)	< 0.001
No	37	26 (70.27%)	11 (29.73%)	

FIGO, International Federation of Gynecology and Obstetrics; OC, ovarian cancer

The enumeration data were analyzed by Chi-squared Test; n = 69; the experiment repeated 3 times, and *p* < 0.05 indicated statistically significant difference

### Silencing of EZH2 inhibits proliferation, migration, invasion and promotes apoptosis in OC cells

**Fig. 3:**
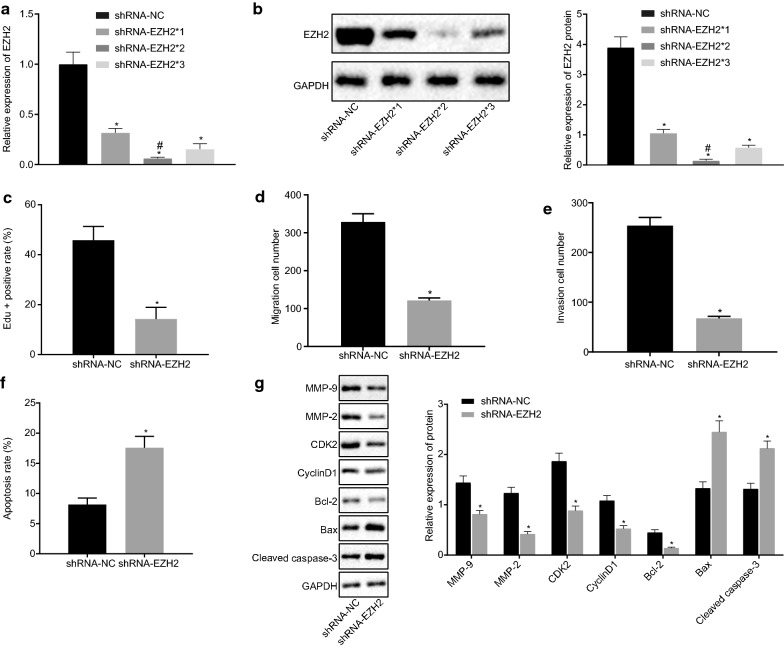
Silencing of EZH2 inhibits proliferation, migration, and invasion, and induces apoptosis in OC cells. **a** The mRNA expression pattern of EZH2 after treatment with shRNA-EZH2*1, shRNA-EZH2*2 and shRNA-EZH2*3 evaluated by RT-qPCR; **b** The protein expression pattern of EZH2 after treatment with shRNA-EZH2*1, shRNA-EZH2*2 and shRNA-EZH2*3 assessed by western blot analysis. In panels C-G, SKOV3 cells were treated with shRNA-NC and shRNA-EZH2 plasmids. **c** The proliferation ability of each group tested by EdU staining (×200). **d** The cell migration ability determined by Transwell assay (×100). **e** The invasive ability of cells in each group examined by Transwell assay (×100). **f** The cell apoptosis in each group measured by flow cytometry. **g** The protein expression pattern of CyclinD1, CDK2, MMP-2, MMP-9, Cleaved Caspase-3, Bax and Bcl-2 in cells assessed by western blot analysis. *p < 0.05 vs. cells transfected with shRNA-NC. The measurement data were expressed as mean ± standard deviation. The paired-designed data was compared using the paired *t*-test. The one-way analysis of variance was adopted for comparison among multiple groups, followed by the Tukey’s post hoc test. The experiment was conducted 3 times independently

Due to the abnormally high expression pattern of EZH2 in OC, we hypothesized that silencing the EZH2 expression pattern could prevent the development of OC. Firstly, three silencing sequences were set, and the silencing efficiency was detected using RT-qPCR and Western blot assay. The expression pattern of EZH2 after transfection with the shRNA-EZH2*2 sequence was the lowest (*p* < 0.05; Fig. 3a, b), hence shRNA-EZH2*2 was selected for further experimentation. Then, the SKOV3 cells were transfected with the shRNA-NC and shRNA-EZH2 plasmids. EdU assay results showed that the SKOV3 cell proliferation ability was reduced upon transfection with shRNA-EZH2 (*p* < 0.05; Fig. 3c). Besides, to detect the migration and invasion ability, Transwell assay was conducted, which revealed that silencing of EZH2 inhibited SKOV3 cell migration and invasion (*p* < 0.05; Fig. 3d, e). Meanwhile, the results of flow cytometry demonstrated that silencing of EZH2 promoted SKOV3 cell apoptosis (*p* < 0.05; Fig. 3f). Furthermore, immunoblotting was employed to detect the protein expression pattern of various cell proliferation- (CyclinD1, CDK2), metastasis- (MMP-2, MMP-9) and apoptosis-related genes (Cleaved Caspase-3, Bax, and Bcl-2). The results revealed that the cells transfected with shRNA-EZH2 showed lower protein expression patterns of CyclinD1, CDK2, MMP-2, MMP-9 and Bcl-2, and higher expression patterns of Cleaved Caspase-3 and Bax (*p* < 0.05; Fig. 3g). In summary, EZH2 silencing inhibits cell proliferative, migratory and invasive abilities and promotes apoptosis.

### EZH2 down-regulates miR-139 expression by recruiting H3K27me3 to promote methylation of miR-139 promoter

**Fig. 4:**
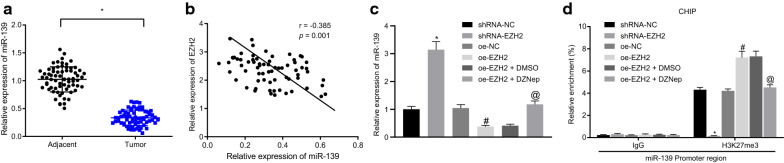
EZH2 down-regulates miR-139 expression through promoting the methylation of miR-139 promoter by recruiting H3K27me3. **a** miR-139 expression pattern in the OC tissues and adjacent normal tissues evaluated by RT-qPCR. **b** The correlation between miR-139 expression pattern and EZH2 expression pattern as assessed by Pearson correlation analysis. In panels C and D, SKOV3 cells were treated with shRNA-EZH2, oe-EZH2 alone or in the presence of DZNep. **c** The miR-139 expression pattern in cells determined by RT-qPCR. **d** The enrichment of H3K27me3 in the miR-139 promoter region of cells measured by ChIP. *p < 0.05 vs. adjacent normal tissues or the shRNA-NC group, ^#^*p* < 0.05 vs. the oe-NC group, ^@^*p* < 0.05 vs. the oe-EZH2 + DMSO group. The measurement data were expressed as mean ± standard deviation. The paired-designed data was compared using paired t-test. The one-way analysis of variance was adopted for comparison among multiple groups, followed by Tukey’s post hoc test. The experiment was conducted 3 times independently

According to a previous research [17], miR-139 was poorly expressed in OC, and the low expression of miR-139 facilitated the progression of various biological processes such as proliferation and metastasis of OC cells, from which it was speculated that miR-139 was a tumor suppressor. RT-qPCR was performed on the collected OC tissues and their adjacent normal tissues, revealing that miR-139 was predominantly expressed in the OC tissues and rarely expressed in the adjacent normal tissues (*p* < 0.05; Fig. 4a). Pearson correlation analysis showed that the miR-139 expression was significantly negatively correlated with the EZH2 expression pattern (*p* < 0.001; Fig. 4b).

Relative literature revealed that EZH2 could down-regulate the miR-139 expression by stimulating miR-139 promoter methylation [18]. To verify this finding, SKOV3 cells were transfected with shRNA-NC, shRNA-EZH2, oe-NC, or oe-EZH2 plasmids, and DMSO and DZNep were added to the cells overexpressing EZH2. RT-qPCR was conducted to measure miR-139 expression pattern after treatment. As anticipated, silencing of EZH2 promoted the miR-139 expression pattern, while overexpression of EZH2 inhibited the miR-139 expression pattern. The miR-139 expression pattern was elevated in cells exposed to the histone methylation inhibitor DZNep (*p* < 0.05; Fig. 4c). These results suggested that EZH2 downregulated miR-139 expression by promoting miR-139 promoter methylation. Further ChIP detection exhibited that the level of H3K27me3 enrichment in the miR-139 promoter region was significantly decreased following EZH2 silencing, while that was significantly increased after EZH2 overexpression. More explicitly, histone methylation inhibitor DZNep significantly reduced the level of H3K27me3 enrichment in the miR-139 promoter region by inhibiting the EZH2 expression pattern (*p* < 0.05; Fig. 4d), implying that miR-139 was regulated by EZH2 and H3K27me3. Collectively, EZH2 promotes the methylation of the miR-139 promoter by incorporating H3K27me3 to down-regulate the miR-139 expression pattern.

### miR-139 inhibits proliferation and migration but promotes apoptosis in OC cells by targeting LPA1

**Fig. 5:**
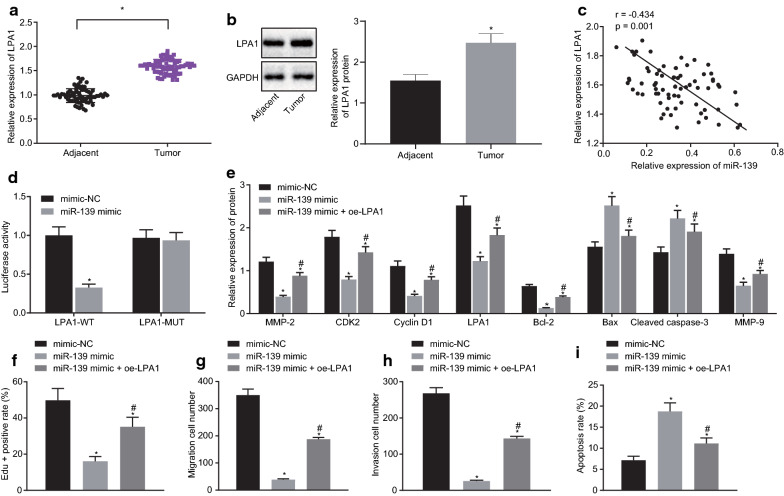
MiR-139 inhibits proliferation and migration of OC cells and promotes apoptosis by repressing LPA1. **a** The mRNA expression pattern of LPA1 in the OC tissues and adjacent normal tissues as determined by RT-qPCR (n = 69). **b** The protein expression pattern of LPA1 in OC tissues and adjacent normal tissues as measured by western blot analysis (n = 69). **c** The correlation between miR-139 expression and LPA1 expression pattern via Pearson correlation analysis. **d** The binding relationship between LPA1 and miR-139 verified by dual-luciferase reporter gene assay. **e**–**i**, SKOV3 cells were treated with miR-139 mimic alone or in the presence of oe-LPA1. **e** The protein expression pattern of CyclinD1, CDK2, MMP-2, MMP-9, Cleaved Caspase-3, Bax, and Bcl-2 in cells as assessed by western blot analysis. **f** Cell proliferation ability detected by EdU assay (×200). **g** Cell migration ability determined by Transwell assay (×100). H, cell invasion ability monitored using Transwell assay (×100). I, cell apoptosis evaluated using Flow cytometry. *p < 0.05 vs. control or mimic NC, ^#^*p* < 0.05 vs. miR-139 mimic group. The measurement data were expressed as mean ± standard deviation. The paired-designed data was compared using paired t-test. The one-way analysis of variance was adopted for comparison among multiple groups, followed by Tukey’s post hoc test. The experiment was conducted 3 times independently

RT-qPCR and Immunoblotting were performed to investigate the LPA1 expression pattern in the collected OC and adjacent normal tissues. The results displayed that the expression of LPA1 in the OC tissues was statistically upregulated compared to the expression in the adjacent normal tissues (*p* < 0.05; Fig. 5a, b). Pearson correlation analysis showed that miR-139 was significantly negatively correlated with LPA1 (*p* < 0.001; Fig. 5c), suggesting that LPA1 may be regulated by miR-139. The dual-luciferase reporter gene assay found that miR-139 could exclusively target and inhibit the LPA1 expression pattern (*p* < 0.05; Fig. 5d), that was, LPA1 may be a putative target of miR-139, which was in consistency with the Pearson correlation analysis.

To investigate the effect of miR-139-mediated LPA1 on the development of OC, SKOV3 cells were transfected with mimic-NC, miR-139 mimic, or co-transfected with miR-139 mimic and oe-LPA1 plasmids. Immunoblotting was subsequently conducted to detect the protein level of LPA1, CyclinD1, CDK2, MMP-2, MMP-9, Cleaved Caspase-3, Bax and Bcl-2. In contrast to the cells without transfection and cells transfected with NC, the cells transfected with miR-139 mimic exhibited reduced LPA1, CyclinD1, CDK2, MMP-2, MMP-9, Bcl-2 protein expression patterns, and elevated protein expression patterns of Cleaved Caspase-3, and Bax (*p* < 0.05; Fig. 5e). Furthermore, the addition of overexpressed LPA1 plasmid elevated the protein expression patterns of CyclinD1, CDK2, MMP-2, MMP-9 and Bcl-2, but reduced the protein expression patterns of Cleaved Caspase-3 and Bax compared to transfection with the miR-139 mimic alone (*p* < 0.05; Fig. 5e). EdU assay results depicted that up-regulation of miR-139 inhibited SKOV3 cell proliferation, and overexpression of LPA1 annulled the result induced by miR-139 mimic (*p* < 0.05; Fig. 5f). Subsequently, the results of Transwell assay illustrated that the treatment of miR-139 mimic inhibited the migration and invasion of SKOV3 cells; while the co-treatment of oe-LPA1 and miR-139 mimic increased the migration and invasion abilities of SKOV3 cells relative to miR-139 mimic treatment (*p* < 0.05; Fig. 5g, h). Additionally, flow cytometry revealed that overexpressed miR-139 promoted SKOV3 cell apoptosis; whereas overexpression of LPA1 resulted in a decline in the apoptosis of miR-139 mimic-treated SKOV3 cells (*p* < 0.05; Fig. 5i). In conclusion, up-regulation of miR-139 inhibits the proliferative, migratory and invasive abilities, and promotes the apoptosis of OC cells.

### Overexpressed miR-139 blocks the TGF-β pathway activation by down-regulating LPA1

**Fig. 6:**
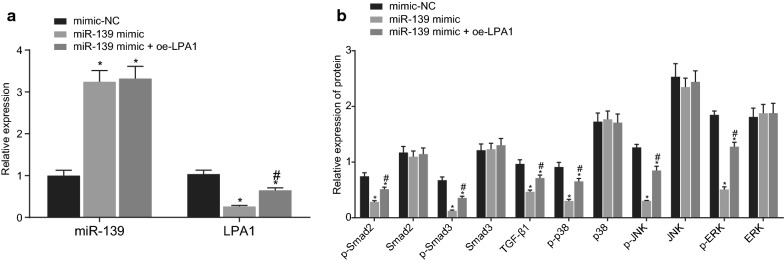
Overexpression of miR-139 blocks the TGF-β signaling pathway activation by down-regulating LPA1. SKOV3 cells were treated with miR-139 mimic alone or in the presence of oe-LPA1. **a** the miR-139 expression pattern and the mRNA expression pattern of LPA1 of cells as determined by RT-qPCR; **b** the protein expression pattern of TGF-β1, Smad3, p-Smad3, Smad2, p-Smad2, ERK, p-ERK, JNK, p-JNK, p38, p-p38 in cells as measured by Western blot analysis; *p < 0.05 vs. mimic NC group, ^#^*p* < 0.05 vs. miR-139 mimic group. The measurement data were expressed as mean ± standard deviation. The one-way analysis of variance was adopted for comparison among multiple groups, followed by Tukey’s post hoc test. The experiment was conducted 3 times independently

The TGF-β pathway is a typical signaling pathway affecting homeostatic cell function, and phosphorylation of the TGF-β signaling pathway-related factors activates the TGF-β pathway and induces tumor metastasis [19, 20]. LPA1 may principally function as a mediator of the TGF-β signal [15]. Since highly expressed LPA1 promotes the migration and invasion of OC cells, we speculated that LPA1 promotes the migration and invasion of OC cells by activation of the TGF-β signaling pathway. Meanwhile, the preceding results identified LPA1 as a target gene of miR-139, and overexpression of miR-139 could reduce the LPA1 expression pattern, so we further speculated that overexpression of miR-139 can hinder the activation of the TGF-β pathway by targeting the LPA1 expression. To verify this inference, SKOV3 cells were transfected with mimic-NC, miR-139 mimic, or co-transfected with miR-139 mimic and oe-LPA1 plasmids, and RT-qPCR assay showed that the overexpression of miR-139 inhibited the LPA1 expression pattern (*p* < 0.05), while LPA1 did not affect the miR-139 expression patterns (*p* > 0.05; Fig. 6a). Immunoblotting was then conducted to detect the expression patterns of various TGF-β pathway-related factors (TGF-β1, Smad3, phosphorylated Smad3, Smad2, phosphorylated Smad2, ERK, phosphorylated ERK, JNK, phosphorylated JNK, p38, phosphorylated p38) after transfection. The expression of Smad3, Smad2, ERK, JNK and p38 were not significantly different among the groups (*p* > 0.05; Fig. 6b). However, the expression pattern of TGF-β1, and the levels of phosphorylated Smad3, phosphorylated Smad2, phosphorylated ERK, phosphorylated JNK and phosphorylated p38 were lowered upon transfection with the miR-139 mimic. Meanwhile, the restoration of LPA1 with the additional usage of oe-LPA1 enhanced TGF-β1, phosphorylated Smad3, phosphorylated Smad2, phosphorylated ERK, phosphorylated JNK, and phosphorylated p38 expression in the OC cells in contrast to the cells transfected with the miR-139 mimic alone (*p* < 0.05; Fig. 6b). In conclusion, overexpression of LPA1 initiates the TGF-β signaling pathway, whereas up-regulation of miR-139 impedes activation of the TGF-β signaling pathway by targeting LPA1.

### **The EZH2/miR-139/LPA1/TGF-β axis is involved in the progression of OC**

**Fig. 7:**
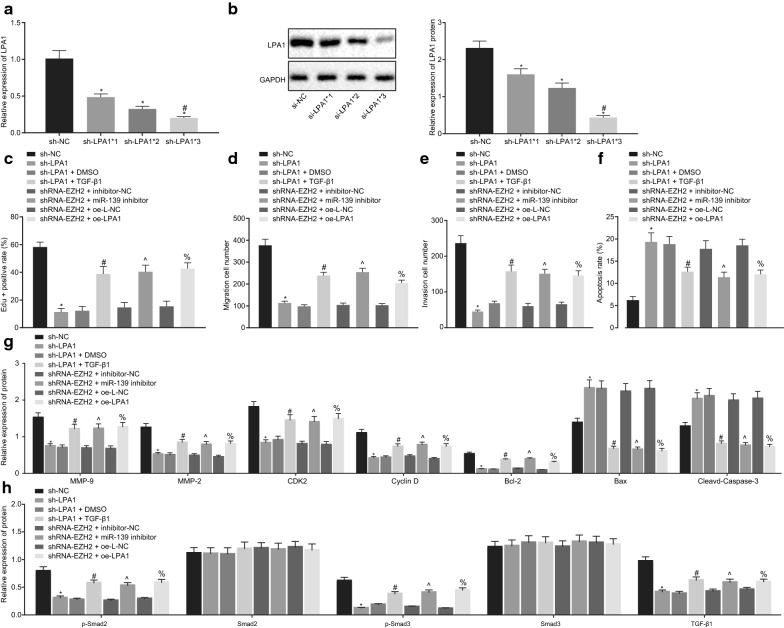
The induction of EZH2, LPA1 and the TGF-β signaling pathway are responsible for accelerated OC cell proliferation, migration, invasion and delayed apoptosis. **a** The mRNA expression of LPA1 after treatment with shRNA-LPA1*1, shRNA-LPA1*2 and shRNA-LPA1*3 evaluated by RT-qPCR; **b** The protein expression pattern of LPA1 after treatment with shRNA-LPA1*1, shRNA-LPA1*2 and shRNA-LPA1*3 assessed by western blot analysis. **c** Cell proliferation ability detected by EdU assay (×200). **d** Cell migration ability determined by Transwell assay (×100). **d** Cell invasive ability examined by Transwell assay (×100). **f** Cell apoptosis monitored by flow cytometry. **g** CyclinD1, CDK2, MMP-2, MMP-9, Cleaved Caspase-3, Bax and Bcl-2 related protein expression pattern examined by western blot analysis. **h** The protein expression pattern of TGF-β signaling pathway-related factors measured by western blot analysis. *p < 0.05 vs. sh-NC group, ^#^*p* < 0.05 vs. sh-LPA1 + DMSO group, ^^^*p* < 0.05 vs. sh-EZH2 + inhibitor NC group, ^%^*p* < 0.05 vs. sh-EZH2 + oe-L-NC group. The measurement data were expressed as mean ± standard deviation. The statistical significance of the data between two groups was calculated using the unpaired *t*-test. The one-way analysis of variance was adopted for comparison among multiple groups, followed by the Tukey’s post hoc test. The experiment was conducted 3 times independently

To investigate the mechanism regarding how the EZH2/miR-139/LPA1 axis affects the development of OC, SKOV3 cells were treated with sh-NC, sh-LPA1, shRNA-EZH2 + inhibitor-NC, shRNA-EZH2 + miR-139 inhibitor, shRNA-EZH2 + oe-L-NC, or shRNA-EZH2 + oe-LPA1 plasmids in combination, and 10 ng/mL of the TGFβ1 agonist and its NC DMSO were also added to the cells transfected with sh-LPA1 for 2 h prior to subsequent experiments. Three silencing sequences were set, and RT-qPCR and Western blot analysis were conducted to detect the silencing efficiency. The results revealed that the expression pattern of LPA1 after transfection with the sh-LPA1*3 sequence was the lowest (*p* < 0.05; Fig. 7a, b), so the sequence of sh-LPA1*3 was selected for subsequent experimentation. EdU assay identified that the knockdown of LPA1 inhibited SKOV3 cell proliferation; addition of TGF-β1 agonist rescued the SKOV3 cell proliferation inhibited by sh-LPA1, and down-regulation of miR-139 or overexpression of LPA1 partially reversed the inhibitory effect of shRNA-EZH2 on SKOV3 cell proliferation (*p* < 0.05; Fig. 7c). Furthermore, the results of Transwell assay showed that knockdown of LPA1 inhibited migration and invasion of the SKOV3 cells. In contrast, the addition of a TGF-β1 agonist restored SKOV3 cell migration and invasion restrained by sh-LPA1, and down-regulation of miR-139 or overexpression of LPA1 annulled the inhibitory effect of shRNA-EZH2 on SKOV3 cell migration and invasion (*p* < 0.05; Fig. 7d, e). The flow cytometry results revealed that the apoptosis of SKOV3 cells was promoted by knockdown of LPA1; the addition of TGF-β1 activation reduced the stimulatory effect induced by sh-LPA1, and down-regulation of miR-139 or overexpression of LPA1 reversed the promotive effect of shRNA-EZH2 on SKOV3 cell apoptosis (*p* < 0.05; Fig. 7f). Immunoblotting results showed that knockdown of LPA1 inhibited CyclinD1, CDK2, MMP-2, MMP-9, Bcl-2, TGF-β1, p-Smad3, p-Smad2 expression patterns and promoted the protein expression patterns of Cleaved Caspase-3 and Bax. On the contrary, addition of a TGF-β1 agonist annulled the alterations in the aforementioned proteins caused by sh-LPA1, and down-regulation of miR-139 or overexpression of LPA1 annulled the alterations in the aforementioned proteins triggered by shRNA-EZH2 (*p* < 0.05; Fig. 7g, h); No difference was evident in the Smad3 and Smad2 expression pattern in response to different treatment protocols (*p* > 0.05; Fig. 7h). Collectively, these results suggested that silencing of EZH2 was a potency to promote miR-139 expression to subsequently inhibit OC cell proliferation, migration, invasion, and promote apoptosis by impeding activation of the LPA1-dependent TGF-β signaling pathway.

### Silencing of EZH2 inhibits tumor growth of OC in nude mice

**Fig. 8:**
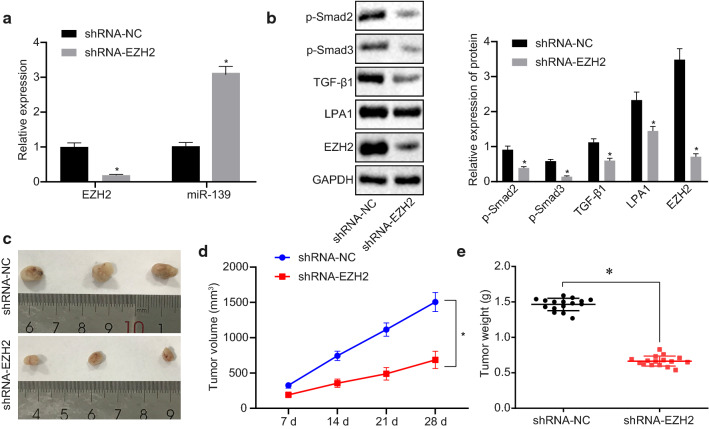
Silencing of EZH2 inhibits tumor growth in vivo by elevating miR-139 expression. The tumor xenograft model was established by injecting the nude mice with the cells stably transfected with shRNA-NC and shRNA-EZH2. **a** The mRNA expression pattern of EZH2 and miR-139 in the tumor tissues of nude mice determined by RT-qPCR. **b** The protein expression patterns of EZH2, LPA1, TGF-β1, p-of Smad3 and p-Smad2 in the tumor tissues of nude mice as measured by western blot analysis. **c** The representative images of tumor in nude mice. **d** The tumor volume in nude mice at the 7th, 14th, 21st, and 28th d after injection. **e** Tumor weight of nude mice. *p < 0.05 vs. mimic-NC group, ^#^*p* < 0.05 vs. miR-139 mimic group. The measurement data were expressed as mean ± standard deviation. The statistical significance of the data (n = 16) between two groups was calculated using the unpaired *t*-test

In the cell experiments, silencing of EZH2 promoted the miR-139 expression pattern to further inhibit OC cell proliferation, migration, invasion, and promote apoptosis by blocking the LPA1-mediated TGF-β signaling pathway activation. Next, we investigated in vivo whether silencing of EZH2 could retard the growth of tumor xenografts in nude mice. The cells stably transfected with shRNA-NC and shRNA-EZH2 were seeded into the nude mice to establish a subcutaneous tumor xenograft model. RT-qPCR results showed that silencing of EZH2 increased the miR-139 expression pattern (*p* < 0.05; Fig. 8a). The result of immunoblotting indicated that the protein expression patterns of EZH2, LPA1 and the TGF-β signaling pathway-related factors (TGF-β1, p-Smad3, p-Smad2) in the nude mice were reduced significantly in the shRNA-EZH2 group relative to the shRNA-NC group (*p* < 0.05; Fig. 8b). In comparison with the shRNA-NC group, the mice in the shRNA-EZH2 group showed significant inhibition of the tumor growth. Additionally, the tumor weight and tumor volume of the nude mice in the shRNA-EZH2 group decreased significantly in contrast to those in the shRNA-NC group (*p* < 0.05; Fig. 8c–e). In conclusion, EZH2 silencing could restore the miR-139 expression pattern, and subsequently inhibit the expression pattern of LPA1 and activation of the TGF-β signaling pathway, thus impeding the growth of OC cells in vivo.

## Discussion

EZH2 frequently functions as a specific H3K27 methyltransferase to intrinsically catalyze histone H3K27me3, which is a vital step of the fundamental biological processes of human cancers, and EZH2 has been largely acknowledged as a promoter of cancer development [21]. In OC, the most lethal gynecological disease, EZH2 also serves as a booster for the invasion and migration of OC cells [22]. Our study demonstrated that EZH2 could accelerate OC progression through the miR-139/LPA1/TGF-β axis.

Initially, EZH2 was identified as a differentially expressed gene on the basis of the OC-related microarray GSE54388, where EZH2 upregulation was identified in OC. In consistency with our findings, overexpressed EZH2 has been previously correlated with shorter survival among OC patients [23], and EZH2 depletion could not only reduce tumor cell proliferation but also suppress the drug resistance in OC [24]. In a previous study, the role of EZH2 promoting cancerous cell proliferation and invasion in ovarian cancer was also been reported [25]. However, it only investigated the expression and function of EZH2 in ovarian cancer, and the underlying mechanism was not discussed. Another study explored the relationship between EZH2 and P53, while our investigation focused on that EZH2 downregulated miR-139 expression to promote the occurrence of ovarian cancer via LPA1 upregulation [26]. Besides, EZH2/H3K27Me3 and phosphorylated EZH2 were reported to predict chemotherapy response and prognosis in ovarian cancer [27]. However, the relationship between EZH2 and H3K27Me3 was only the theoretical basis for the mechanism of EZH2 regulating miR-139 not the main focus or novelty of our investigation. Therefore, although the above-mentioned articles reported about EZH2, our study initially investigated the interaction among EZH2, miR-139, and LPA1, and explored the function and underlying mechanism of EZH2 in ovarian cancer. It provides a novel possible mechanism for ovarian cancer treatment. Our findings elicited that EZH2 was associated with the histological grade, the FIGO stage and LNM in OC patients, and a higher EZH2 expression was correlated with poor prognosis. Moreover, the silencing of EZH2 inhibited OC cell proliferation, migration and invasion, but stimulated cell apoptosis.

The regulatory relationship between EZH2 and miR-139 was then identified in the present study. EZH2 regulating miR-139 has been reported in various diseases; for instance, EZH2 exercises its pro-metastatic role by suppressing miR-139-5p expression in hepatocellular carcinoma [16]. However, in light of the previous literature, atypical studies elucidated the relationship between EZH2 and miR-139 in OC. MiR-139 was downregulated in epithelial OC and therefore considered as a viable therapy for OC as it could suppress the proliferation, migration and invasion of tumor cells [28]. A previous study has reported that miR-139 inhibits OC growth and metastasis via ELAVL1 [11], yet our study has revealed a new regulatory axis that miR-139 targets LPA1 to suppresses OC progression for the first time. Moreover, we also verified that miR-139 could be regulated by its upstream gene EZH2 through the methylation of miR-139 promoter. An existing study identified the ability of EZH2 to repress miR-708 expression by incorporating promoter methylation in the glioma tissues and cells [29]. Likewise, the miR-211 expression was reduced by EZH2-mediated methylation in malignant melanoma [7], and overexpression of EZH2 significantly decreased miR-193a expression in OC [30], which was in compliance with the observed mechanism of EZH2-mediated methylation in our study. Our findings elicited that EZH2 could downregulate the miR-139 expression by engaging H3K27me3 to stimulate the methylation of miR-139 promoter, as proved by the ChIP assay. Notably, inhibition of EZH2 upon instilling DZNep treatment could reduce the survival of epithelial OC cells *by* potentiating the expression pattern of aplysia ras homolog member I, a tumor-suppressor gene [31].

Upon elucidating the upstream regulatory mechanism of miR-139, we further explored the downstream mechanism of miR-139, thereby unraveling LPA1 as a target gene of miR-139. A prior work declared that LPA1 could facilitate OC cell migration and invasion and promote tumor growth in vivo [32], and it was responsible for the metastasis of OC [33]. Interestingly, miR-139-5p was identified to target a key member of the LPA receptor family, LPA receptor 4 in interstitial cystitis [34]. To the best of our knowledge, we initially stated that in OC, LPA1 was the viable target gene of miR-139, and hence it was negatively regulated by miR-139. In light of the preceding finding, upregulated miR-139 could terminate the progression of OC by inhibiting LPA1 expression. Furthermore, LPA1 mediating the TGF-β signaling pathway was investigated in our study, in which a positive correlation between them was identified. Numerous studies have demonstrated the involvement of the TGF-β signaling pathway in OC [35, 36]. Besides, TGF-β was corroborated to lower the LPA1 expression and reduce LPA1-dependent cell migration and invasion in a Smad-dependent manner [37]. Furthermore, an association between LPA1 and TGF-β-induced pulmonary fibrosis has been previously observed [38]. This may be rationalized based on the synchronous mechanism that LPA1 coupled to ERK1/2 signaling, thereby contributing to ERK1/2-dependent elevation in TGF-β expression [39]. In our study, miR-139 downregulated LPA1 and consequently hindered the activation of the TGF-β signaling pathway due to the capacity of overexpressed LPA1 to reverse the downregulation of TGF-β1 as well as the subsequent reductions in phosphorylated Smad3, phosphorylated Smad2, phosphorylated ERK, phosphorylated JNK and phosphorylated p38 expression pattern consequent of the miR-139 mimic. In addition to the previously reported role of LPA1-mediated TGF-β, our study substantiated that TGF-β1 activation could inverse the tumor-promotive effect of LPA1, thereby establishing an inductive role of LPA1 in the TGF-β signaling pathway. Moreover, the TGF-β signaling pathway and its related mechanism are being investigated for the development of more targeted OC therapies [40], and our study was one of the researches demonstrating that the EZH2-mediated miR-139 knockdown promoted LPA1 expression to activate the TGF-β signaling pathway, thereby accelerating the OC progression.

## Conclusions

**Fig. 9:**
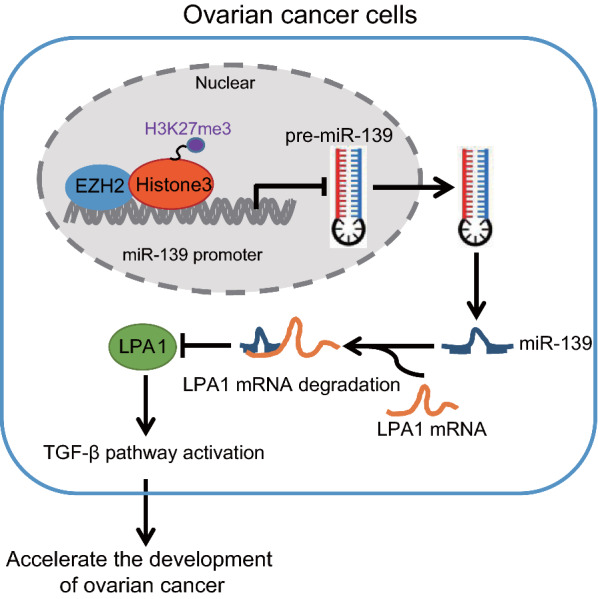
The graphical summary of the function and mechanism of EZH2/miR-139 axis in OC. EZH2 down-regulates the miR-139 expression pattern by recruiting H3K27me3 to promote the miR-139 promoter methylation and activate the TGF-β pathway by up-regulating LPA1, which contributed to the progression of OC

Our study offered paramount evidence elucidating the relationship between miR-139/LPA1 and LPA1/TGF-β in OC, and the candidate target miR-139 for the treatment of OC was described (Fig. 9). Although the limitations in clinical observations due to the experimental conditions, this study appropriately proved the essential roles of EZH2 and EZH2-mediated miR-139 in OC progression, which provided a promising target for OC therapy.

## Data Availability

The datasets generated and/or analyzed during the current study are available from the corresponding author on reasonable request.

## Abbreviations

OC: Ovarian cancer
EZH2: Enhancer of zeste homolog 2
lncRNA: Long non-coding RNA
miR-211: microRNA-211
LAP1: Lysophosphatidic acid
TGF-β: Transforming growth factor beta
GEO: Gene Expression Omnibus
OS: Overall survival
NC: Negative control
RT-qPCR: Reverse transcription quantitation-polymerase chain reaction
GAPDH: Glyceraldehyde-3-phosphate dehydrogenase
BCA: Bicinchoninic acid
WT: Wild type
UTR: Untranslated region
RLU: Relative light unit
ChIP: Chromatin immunoprecipitation
EdU: 5-ethynyl-2′-deoxyuridine
FITC: Fluorescein isothiocyanate
PI: Propidium iodide
HEPES: N-2-hydroxyethylpiperazine-N′-2-ethanesulfonic acid
ANOVA: Analysis of variance
LNM: Lymph node metastasis
