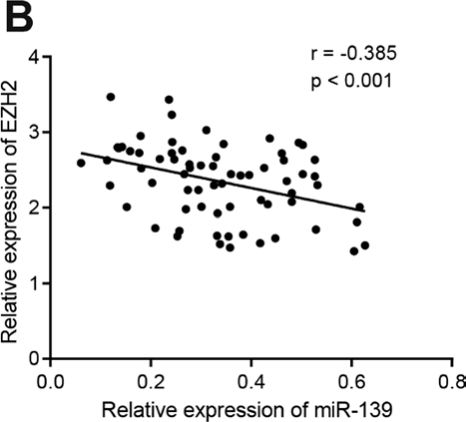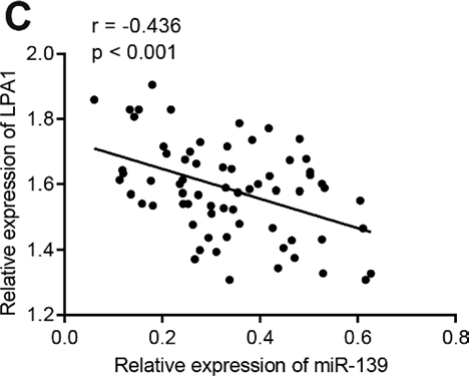# Correction to: EZH2 promotes the expression of LPA1 by mediating microRNA-139 promoter methylation to accelerate the development of ovarian cancer

**DOI:** 10.1186/s12935-021-02236-9

**Published:** 2021-10-19

**Authors:** Dongbo Wu, Fanglan Wu, Birong Li, Wei Huang, Donglian Wang

**Affiliations:** 1 Department of Obstetrics and Gynecology, The First Hospital of Changsha, Changsha, 410000 People’s Republic of China; 2 Department of Clinical Laboratory, The First Hospital of Changsha, Changsha, 410005 People’s Republic of China; 3 Department of Gynecology, Hunan Provincial People’s Hospital, (The First Afliated Hospital of Hunan Normal University), No. 61, Western Jiefang Road, Changsha, 410000 Hunan People’s Republic of China; 4 Department of Gynecology, The Maternal and Child Health Hospital of Hunan Province, Changsha, 410000 People’s Republic of China

## Correction to: Cancer Cell Int (2020) 20:551 10.1186/s12935-020-01622-z

Following the publication of the original article [1], we were notified that due to unskilled software and analysis, the correlation results in Figs. 4B and 5C were incorrect. As the software can now directly calculate the fitting curve, the correct analysis results are given below: